# Efficacy of Adjunct PRObiotics as Compared to the Standard Care in Moderate Unipolar Depression Among Geriatric Patients: A Randomized Double‐Blind Placebo‐Controlled Pilot Multi‐Center Trial (PRODG)

**DOI:** 10.1111/jgs.70530

**Published:** 2026-06-17

**Authors:** Preeti Sinha, Prasun Chatterjee, Preethy Kathiresan, Karishma Sundara Raju, Rasika Panwar, Aparna Mukherjee, Gunjan Kumar, Jerin Jose Cherian, Anoop Velayuthan, Avinash Chakrawarty, Sarnendu Mondal, Manoj Kalita, Spriha Kamboj, Sreyashi Sen, Mounamukhar Bhattacharjee, Manaswini Mondal, Kalyan Bhowmik, Sovonlal Mukherjee, Indranil Saha, Atanu Kumar Dutta, Asim Saha, Amit Chakrabarti, Abhinaba Ghosh, Saibal Das

**Affiliations:** ^1^ Department of Psychiatry National Institute of Mental Health and Neurosciences Bengaluru India; ^2^ Department of Geriatric Medicine All India Institute of Medical Sciences New Delhi India; ^3^ Department of Psychiatry All India Institute of Medical Sciences New Delhi India; ^4^ Indian Council of Medical Research New Delhi India; ^5^ Academy of Scientific and Innovative Research (An Institution of National Importance Established by an Act of Parliament) Ghaziabad India; ^6^ Department of Global Public Health Karolinska Institutet Stockholm Sweden; ^7^ Indian Council of Medical Research – National Institute for Research in Bacterial Infections Kolkata India; ^8^ Indian Council of Medical Research – Regional Medical Research Centre North‐East Dibrugarh India; ^9^ Department of Biochemistry All India Institute of Medical Sciences Kalyani India; ^10^ Indian Council of Medical Research – Centre for Ageing and Mental Health Kolkata India; ^11^ Tata Medical Centre Kolkata India

**Keywords:** anxiety, depression, geriatric, gut‐brain axis, older, probiotics

## Abstract

**Objective:**

To evaluate the efficacy of adjunct probiotic supplementation ( *Lactobacillus helveticus* and *Bifidobacterium longum* ) alongside standard care compared to placebo in older adults with moderate unipolar depression.

**Methods:**

A randomized, double‐blind, placebo‐controlled pilot trial was conducted at two tertiary centers. Fifty‐eight participants (≥ 60 years) with moderate depression were randomized 1:1 to receive daily probiotics or a placebo for 12 weeks, alongside standard antidepressant care. They were followed up for another 12 weeks. The primary outcome was depression response (≥ 50% Montgomery‐Åsberg Depression Rating Scale [MADRS] score reduction). Secondary outcomes included anxiety (General Anxiety Disorder 7‐Item [GAD‐7]), cognition, quality of life (WHOQOL‐BREF), serum brain‐derived neurotropic factor (BDNF), and gut microbiota profile.

**Results:**

Mixed‐effects models showed significant improvement over time in depressive symptoms (MADRS: *F* = 32.0, *p* < 0.001) and anxiety (GAD‐7: *F* = 13.1, *p* < 0.001). Overall scores were lower in the probiotic group compared with the placebo group for both MADRS (*F* = 12.7, *p* = 0.001) and GAD‐7 (*F* = 10.7, *p* = 0.002), although group × time interactions were not significant. Quality‐of‐life domains improved markedly (all *F* > 100, *p* < 0.001) without additional benefit from probiotics. Escitalopram‐equivalent antidepressant dose and benzodiazepine use influenced selected outcomes. The probiotic group also had a significantly higher serum BDNF level and increased fecal abundance of supplemented strains vs. the placebo group. The attrition rate was > 50% over 24 weeks.

**Conclusion:**

In this pilot PRODG trial, adjunct probiotics produced modest overall advantages for depressive and anxiety symptoms compared with placebo but did not enhance quality‐of‐life beyond usual improvement − both groups improved substantially, and trajectories over 24 weeks were largely parallel across follow‐up.

## Summary

- Key points
  ○ Adjunctive probiotics (*Lactobacillus helveticus* and *Bifidobacterium longum*) showed modest improvements in depressive and anxiety symptoms among older adults with moderate depression.
  ○ Probiotic supplementation significantly increased serum BDNF levels and fecal abundance of supplemented strains, supporting a biologically plausible gut‐brain mechanism.
  ○ Despite symptomatic benefits, probiotics did not significantly enhance quality of life beyond standard care, and findings were limited by high attrition and treatment heterogeneity.
- Why does this paper matter?
  ○ This pilot trial provides early evidence that probiotics may serve as a safe, biologically plausible adjunctive therapy for geriatric depression, particularly relevant for low‐resource and aging populations like India.

## 1 Introduction

Depression in older adults is a common and debilitating condition. In older adults, the prevalence of depressive disorders is about 35.1% (95% CI: 30.2 to 40.4%), increasing with advancing age [1]. Standard antidepressant treatments often have limited effectiveness in this population, with lower response rates (around 50% in older patients) [2, 3] and a high placebo response, alongside greater risks of adverse effects [4]. There is a pressing need for novel adjunct therapies to improve outcomes for geriatric depression.

The gut‐brain axis is a complex, bidirectional communication network involving the central nervous system, immune system, and gut microbiota [5, 6]. Gut microbes can influence brain function by producing neuroactive compounds such as serotonin and gamma‐aminobutyric acid, which act on the vagus and enteric nerves [5, 6, 7]. Stress and hypothalamic–pituitary–adrenal axis activation can disrupt gut barrier integrity and alter microbiota composition, contributing to psychiatric conditions like depression and anxiety [8]. Probiotic supplementation offers a potential therapeutic approach by restoring microbial balance and modulating brain function [9]. Specifically, *Lactobacillus helveticus* and *Bifidobacterium longum* have been shown to improve mood and reduce anxiety in both preclinical models and clinical studies [10, 11, 12, 13, 14, 15, 16, 17]. These probiotics enhance brain‐derived neurotrophic factor (BDNF) levels and support neural resilience [18]. Studies across various countries affirm the safety and efficacy of these strains, suggesting their potential as adjunctive treatments for mental health conditions across diverse populations [10, 11, 12, 13, 14, 15, 16].

Data on probiotic efficacy specifically in older patients with clinical depression remain scarce. Hence, this pilot trial was conducted to evaluate the efficacy of adjunctive probiotic therapy combined with standard care as compared to standard care alone in older patients with moderate unipolar depression. We hypothesized that adding probiotics to standard treatment would improve depression outcomes and related clinical measures relative to standard care with placebo.

## 2 Patients and Methods

### 2.1 Study Design and Setting

The PRODG trial was a multi‐center, randomized, double‐blind, placebo‐controlled parallel‐group pilot trial. It was conducted at two tertiary care hospitals in India (All India Institute of Medical Sciences, New Delhi and National Institute of Mental Health and Neurosciences, Bengaluru) between 2023 and 2025. Older adult patients were allocated 1:1 to receive either an adjunct probiotic plus standard depression care or a placebo plus standard care. The trial was designed as a pilot to assess feasibility and preliminary efficacy; no changes to methods occurred after trial commencement.

### 2.2 Participants

Patients of either sex aged ≥ 60 years with a diagnosis of unipolar major depressive disorder (single or recurrent episode) of moderate severity were recruited. Moderate depression was defined as a current depressive episode lasting ≥ 2 weeks with a Montgomery–Åsberg Depression Rating Scale (MADRS) [19, 20] score of 20–34 at baseline. All participants were either antidepressant‐naïve for the current episode or had a washout of prior antidepressant therapy, so that standard care treatment could be initiated. Key exclusion criteria included: presence of psychotic symptoms or high suicide risk (e.g., active suicidal ideation requiring urgent intervention according to the Beck Scale for Suicidal Ideation [21, 22]), significant cognitive impairment (score < 25 on the Montreal Cognitive Assessment [MoCA] [23, 24]), chronic neurological disorders (e.g., dementia, Parkinson's), active substance abuse, and any gastrointestinal condition (such as irritable bowel syndrome or malabsorption) that could affect probiotic ingestion or efficacy. Patients with uncontrolled medical illnesses or those on immunosuppressive therapy were also excluded.

### 2.3 Randomization and Blinding

Block randomization (of unequal block sizes) was used to assign participants in a 1:1 ratio to the two study groups. The random allocation sequence was computer‐generated by an independent statistician, stratified by two study sites. Allocation concealment was maintained through sequentially numbered, opaque, sealed envelopes prepared off‐site. The trial was double‐blind—neither the participants nor the treating clinicians, outcome assessors, or data analysts were aware of group assignments. The probiotic and placebo capsules were identical in appearance (size, color, and packaging) and flavor/smell. An independent pharmacy service coded and dispensed the study capsules according to the randomization list. Blinding was maintained until all data were collected and the protocol‐specified analyses were completed.

### 2.4 Interventions

All participants received standard antidepressant treatment as per current guidelines for moderate depression. Psychosocial support or psychotherapy was provided as needed as part of usual care, but no structured psychotherapy was mandated. Concomitant anxiolytics (e.g., low‐dose benzodiazepines) were permitted for acute anxiety or insomnia, but use of other probiotics, prebiotics, or any investigational treatments was prohibited. Medication adherence was monitored by pill count. Patients in the probiotics group received an oral probiotic supplement daily for 12 weeks in addition to standard care. The probiotic was provided in capsule form, containing at least 3 billion colony‐forming units (CFU) each of two strains: *L. helveticus* and *B. longum* . These specific strains were chosen based on prior evidence of beneficial effects on mood and anxiety [13, 16]. The total dose was ~6 billion CFU per day. Participants were instructed to take one capsule after dinner. The probiotic capsules were manufactured under Good Manufacturing Practice conditions and packaged in identical bottles and supplied by Genprotic Biopharma Pvt. Ltd., Chennai, India. Patients randomized to the control group received a matching placebo capsule daily for 12 weeks, alongside the same standard antidepressant care. The placebo capsules were indistinguishable from the probiotic (containing inert excipients with no live cultures). Like the probiotic, the placebo was taken once daily. All other aspects of clinical care, including antidepressant management and follow‐up schedule, were identical between the two groups.

### 2.5 Outcomes

The study outcomes are enumerated below. There was no change to trial outcomes after the trial commenced. The primary outcome was the depression treatment response rate at 12 weeks. Response was defined as a ≥ 50% reduction in the MADRS total score from baseline [25, 26]. Secondary outcomes: Secondary endpoints included anxiety improvement (General Anxiety Disorder 7‐Item [GAD‐7] [27]), cognitive changes (MoCA), quality of life (QoL) (World Health Organization's Quality of Life—Brief Version [WHOQOL‐BREF] [28, 29]—appropriately translated and normalized to a score of 100 for each of the following domains: physical, psychological, environmental, and social), serum BDNF levels, gut microbiota composition in a subset of patients, and adverse events. For the estimation of serum BDNF levels, peripheral blood samples were collected in the fed state after resting for 15 min. The serum was separated and stored at −80°C until analysis by conventional enzyme‐linked immunosorbent assay [30]. Fecal microbiota profiling was performed for four random patients in each group using whole‐genome metagenomic sequencing (NovaSeq 151 bp), followed by quality filtering, adapter trimming, and removal of human reads. The relative abundance of *L. helveticus* and *B. longum* was calculated by normalizing mapped read counts to total microbial reads [31, 32].

### 2.6 Sample Size Calculation

This study was designed as a pilot randomized controlled trial to generate preliminary data on the efficacy of probiotic supplementation in reducing depressive symptoms and improving QoL among older adults. As is standard in exploratory research, no formal power calculation was performed [33]. Instead, the sample size was guided by expected differences in proportions of clinical responders at 12 weeks (defined as ≥ 50% reduction in MADRS scores from baseline) between the two groups. The target sample size was 80; however, 58 could be achieved within the stipulated timeframe.

### 2.7 Statistical Analysis

Intention‐to‐treat analysis with imputation could not be performed because of substantial missing data [34]. To account for repeated measurements and for potential confounding clinical factors, exploratory linear mixed‐effects models, with random intercepts for participants and a diagonal covariance structure for the repeated time points, were conducted. Fixed effects of treatment group (probiotics vs. placebo), time (baseline, 6, 12, and 24 weeks), and their interaction were evaluated, along with the presence of comorbidities (hypertension and diabetes mellitus), socioeconomic status, benzodiazepine use, and escitalopram equivalent antidepressant dose. Given that the duration of illness behaved as an unstable covariate and did not contribute meaningfully to model fit, it was omitted from final models. Individual models were fitted for MADRS, GAD‐7, and each of the WHOQOL‐BREF domains. Satterthwaite's approximation was used for the denominator degrees of freedom, while the Bonferroni adjustment was applied to pairwise comparisons. A two‐sided *p*‐value < 0.05 was considered significant. All analyses were conducted using SPSS version 21 (IBM, Armonk, NY).

## 3 Results

**FIGURE 1:**
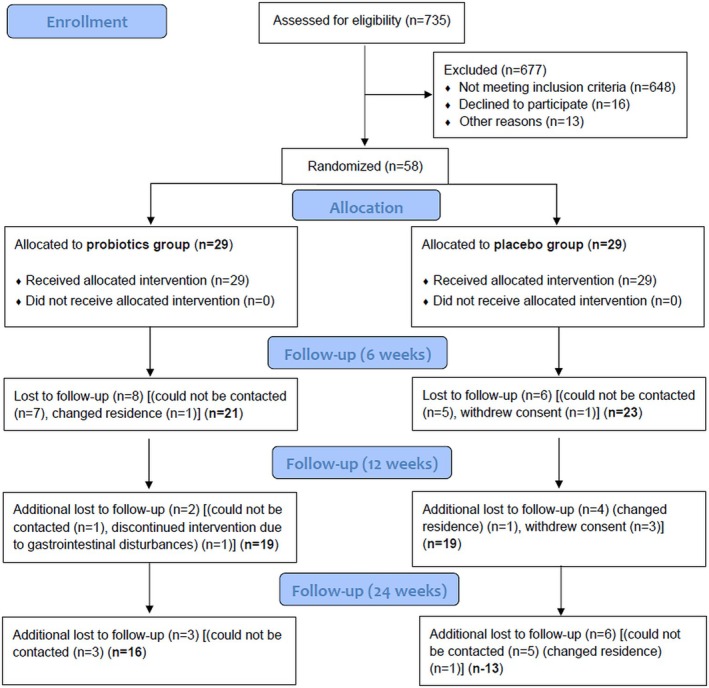
CONSORT flow‐chart of the study participants.

A total of 735 participants were assessed for eligibility, of whom 58 were randomized equally into probiotics and placebo groups. All participants received the allocated intervention. Till 6 weeks, eight and six patients, till 12 weeks, 10 and 10 patients, and till 24 weeks, 13 and 16 patients were lost to follow‐up in the probiotics and placebo groups, respectively (Figure 1). The medication adherence was > 80% in both groups. The two groups were comparable at baseline in terms of demographic characteristics, occupational status, per capita income, and medical/psychiatric comorbidities (Table 1). Standard care was continued as per the treating clinician's discretion. At baseline, there were no statistically significant differences between the probiotic and placebo groups in any of the assessed parameters, confirming adequate comparability of the two groups.

**TABLE 1:** Baseline characteristics of the study patients (*n* = 58).

Parameter	Probiotics group (*n* = 29)	Placebo group (*n* = 29)
Age (years)	65.0 ± 0.7	67.0 ± 0.8
Weight (kg)	60.8 ± 1.7	64.8 ± 1.7
Gender (male)	16 (55.2%)	18 (62.1%)
Occupation		
Homemaker	7 (24.1%)	9 (31.0%)
Retired from service	6 (20.7%)	7 (24.1%)
Farmer	5 (17.2%)	4 (13.8%)
Daily wage worker	4 (13.8%)	3 (10.3%)
Others	7 (24.1%)	6 (20.7%)
Per capita monthly income (INR)		
< 5000	3 (10.3%)	3 (10.3%)
5001–10,000	7 (24.1%)	6 (20.7%)
10,000–20,000	12 (41.4%)	13 (44.8%)
> 20,000	7 (24.1%)	7 (24.1%)
Duration of depression (months)	10.0 ± 3.8	10.0 ± 4.6
Beck's scale of suicidal ideation	4.1 ± 0.4	4.2 ± 0.4
Concomitant psychiatric disorder		
Anxiety disorder	6 (20.7%)	7 (24.1%)
Psychosis	3 (10.3%)	2 (6.9%)
Psychiatric medications		
Sertraline	9 (31%)	15 (51.7%)
Escitalopram	3 (10.3%)	2 (6.9%)
Mirtazapine	2 (6.9%)	1 (3.4%)
Zolpidem	0 (0%)	2 (6.9%)
Concomitant medical disorder		
Hypertension	18 (62.1%)	19 (65.5%)
Dyslipidemia	16 (55.2%)	11 (37.9%)
Diabetes mellitus	15 (51.7%)	16 (55.2%)
Chronic obstructive pulmonary disease	3 (10.3%)	5 (17.2%)
Hypothyroidism	2 (6.9%)	3 (10.3%)
Gastroesophageal reflux disease	5 (17.2%)	2 (6.9%)

*Note:* The results are represented by the mean ± standard error of the mean or number (percentage).

**FIGURE 2:**
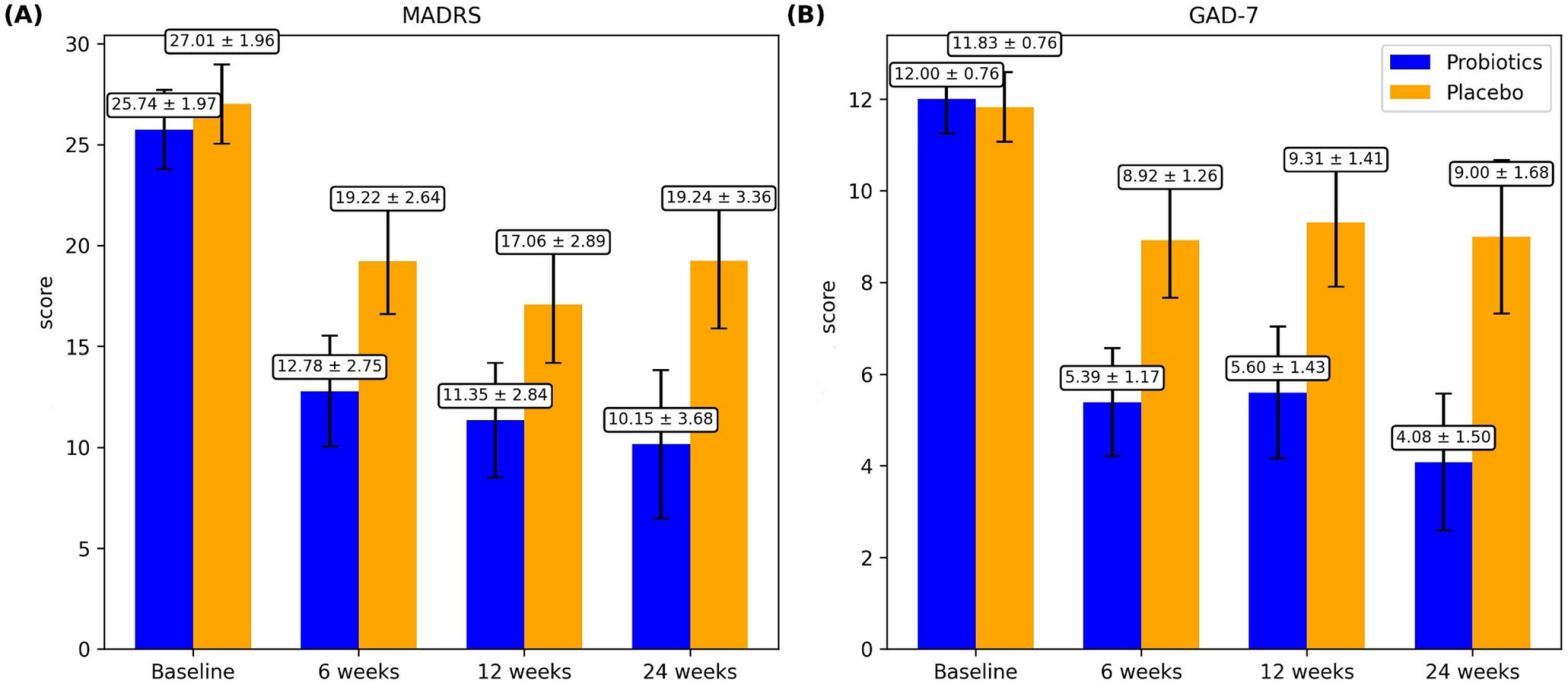
Montgomery–Åsberg depression rating scale (MADRS) score (A) and general anxiety disorder 7‐item (GAD‐7) score (B) in the two groups at different time points (per‐protocol analysis). The results are represented by the mean ± standard error of the mean.

The results of the linear mixed model analysis are enumerated in Table 2. For depressive symptoms (MADRS), there was a robust main effect of time, indicating significant improvement across the 24‐week follow‐up (*F* = 32.0, *p* < 0.001) (Figure 2A). A significant main effect of treatment group was also observed (F = 12.7, *p* = 0.001), indicating overall differences between the probiotic and placebo groups across time. The treatment group × time interaction was not statistically significant (*F* = 2.0, *p* = 0.137), suggesting broadly parallel trajectories of improvement in both groups rather than divergent patterns over time. Among covariates, higher escitalopram equivalent antidepressant dose was a significant predictor of MADRS scores (*F* = 4.5, *p* = 0.037), whereas hypertension, diabetes mellitus, socioeconomic status, and benzodiazepine use were not independently associated with depressive symptom trajectories. For anxiety (GAD‐7), there was again a strong main effect of time (*F* = 13.1, *p* < 0.001), consistent with decreasing anxiety scores over follow‐up (Figure 2B). The treatment group remained a significant predictor of anxiety (*F* = 10.7, *p* = 0.002), indicating overall between‐group differences. The group × time interaction did not reach conventional significance (*F* = 2.4, *p* = 0.075). Benzodiazepine use showed a significant association with GAD‐7 scores (*F* = 12.7, *p* = 0.001), while other covariates, including escitalopram equivalent antidepressant dose, hypertension, diabetes mellitus, and socioeconomic status, were not significant predictors in the multivariable model. The MoCA scores remained relatively stable throughout the study period in both groups.

**TABLE 2:** Linear mixed model analysis of the effects of different factors on depression, anxiety, and quality of life (*n* = 58).

Variable	MADRS		GAD‐7		WHOQOL‐BREF physical		WHOQOL‐BREF psychological		WHOQOL‐BREF environmental		WHOQOL‐BREF social	
	*F*	*p*	*F*	*p*	*F*	*p*	*F*	*p*	*F*	*p*	*F*	*p*
Intercept	55.3	< 0.001*	117.4	< 0.001*	284.3	< 0.001*	974.4	< 0.001*	1187.4	< 0.001*	87.6	< 0.001*
Treatment group^a^	12.7	0.001*	10.7	0.002*	0.1	0.701	3.8	0.059	0.9	0.333	1.5	0.226
Number of weeks^b^	32.0	< 0.001*	13.1	< 0.001*	239.6	< 0.001*	288.6	< 0.001*	107.3	< 0.001*	102.7	< 0.001*
Treatment group × number of weeks	2.0	0.137	2.4	0.075	0.1	0.934	1.5	0.229	0.6	0.617	1.2	0.339
Hypertension (comorbidity)	1.1	0.289	0.8	0.376	0.4	0.516	1.3	0.266	3.9	0.052	16.5	< 0.001*
Diabetes mellitus (comorbidity)	1.6	0.210	3.4	0.066	0.1	0.771	0.1	0.709	0.6	0.427	0.2	0.697
Benzodiazepine use	1.5	0.221	12.7	0.001*	4.6	0.035*	0.6	0.428	0.3	0.590	5.7	0.019*
Escitalopram equivalent antidepressant dose	4.5	0.037*	0.6	0.428	0.0	0.867	2.9	0.093	2.3	0.132	1.0	0.332
Socioeconomic status	0.8	0.363	0.5	0.470	1.2	0.269	0.0	0.970	0.2	0.641	0.1	0.711

Abbreviations: GAD‐7, General Anxiety Disorder 7‐Item; MADRS, Montgomery‐Åsberg Depression Rating Scale; MoCA, Montreal Cognitive Assessment; WHOQOL‐BREF, World Health Organization's Quality of Life—Brief Version.

^a^ Probiotics and placebo groups.

^b^ Baseline, after 6 weeks, after 12 weeks, and after 24 weeks.

* *p* < 0.05.

**FIGURE 3:**
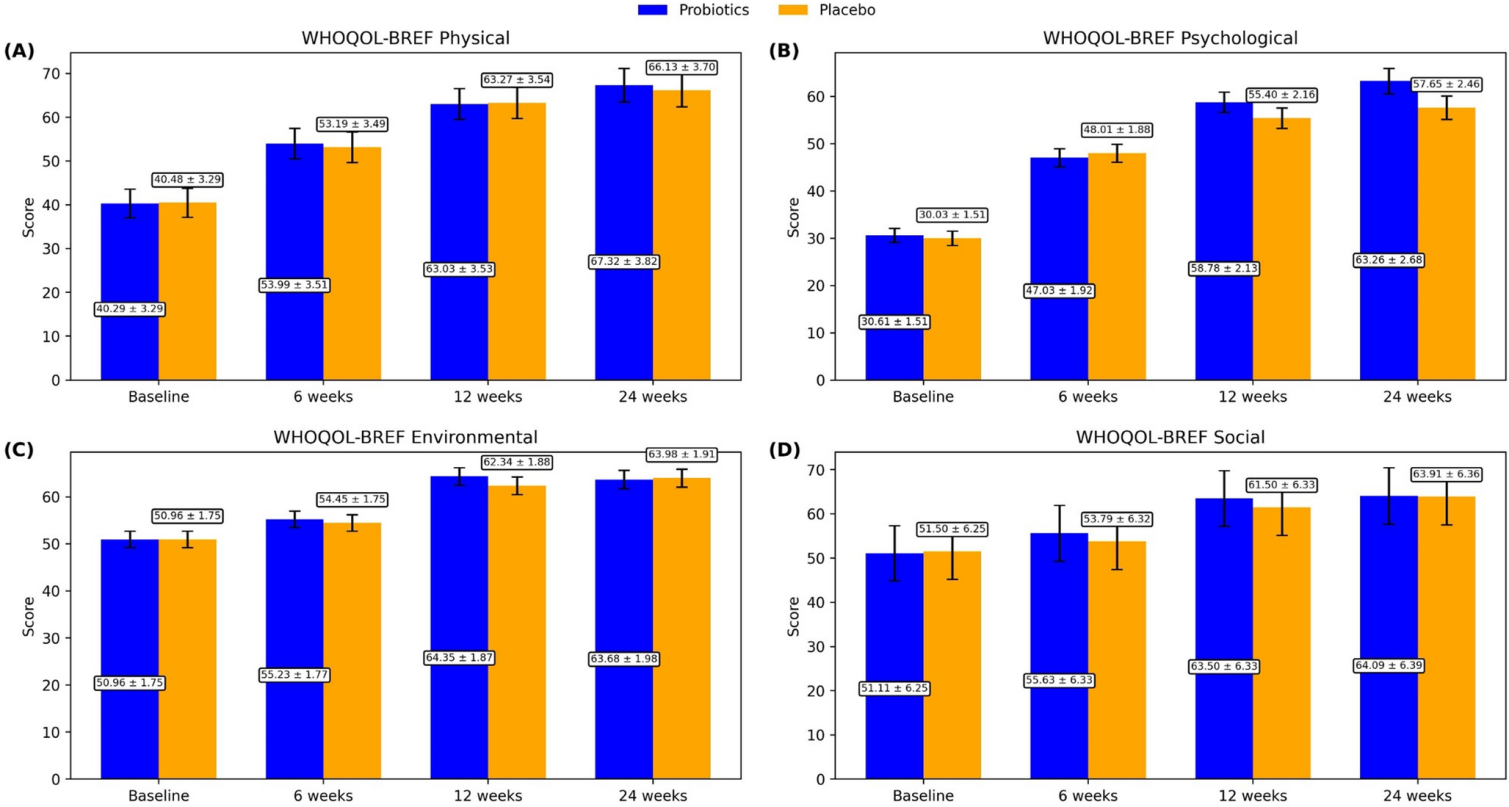
Changes in the World Health Organization's quality of life—brief version (WHOQOL‐BREF) scores (normalized to a score of 100) for each of the four domains, physical (A), psychological (B), environmental (C), and social (D), in the two groups at different timepoints (per‐protocol analysis). The results are represented by the mean ± standard error of the mean.

All four quality‐of‐life domains showed marked improvement over time. Significant main effects of time were observed for physical (*F* = 239.6, *p* < 0.001) (Figure 3A), psychological (*F* = 288.6, *p* < 0.001) (Figure 3B), environmental (*F* = 107.3, *p* < 0.001) (Figure 3C), and social (*F* = 102.7, p < 0.001) (Figure 3D) QoL, indicating better perceived functioning and well‐being across the 24‐week period. In contrast, the main effects of treatment group were not statistically significant for physical (*F* = 0.1, *p* = 0.701), environmental (*F* = 0.9, *p* = 0.333), or social (*F* = 1.5, *p* = 0.226) domains, and showed only a statistical trend for the psychological domain (*F* = 3.8, *p* = 0.059). No significant treatment group × time interactions were detected for any quality‐of‐life outcome (all *p* > 0.22), indicating similar patterns of QoL improvement in both groups. Regarding covariates, hypertension was strongly associated with social quality‐of‐life scores (*F* = 16.5, *p* < 0.001) and showed a near‐significant association with environmental QoL (*F* = 3.9, *p* = 0.052). Benzodiazepine use was significantly related to physical (*F* = 4.6, *p* = 0.035) and social (*F* = 5.7, *p* = 0.019) quality‐of‐life domains, while diabetes mellitus and socioeconomic status were not significant predictors of any quality‐of‐life outcome (all *p* > 0.26).

For the covariates, escitalopram equivalent antidepressant dose was found to have a small but significant relationship with depressive symptoms, where each one‐unit increase in escitalopram equivalent antidepressant dose was related to a 0.32‐point increase in MADRS scores (*β* = 0.32, SE = 0.15, 95% CI: 0.02 to 0.61, *p* = 0.037). Benzodiazepine use was found to be strongly related to higher levels of anxiety, with GAD‐7 scores 3.15 points higher in users compared with non‐users (*β* = 3.15, SE = 0.88, 95% CI: 1.41 to 4.88, *p* < 0.001). Benzodiazepine use was also found to be related to poorer WHOQOL‐BREF items in the Physical (*β* = −1.77, SE = 0.83, 95% CI: −3.41 to −0.15, *p* = 0.035) and Social (*β* = −1.42, SE = 0.59, 95% CI: −2.58 to −0.26, *p* = 0.019) domains. Hypertension showed a small but significant negative relationship with WHOQOL‐BREF items in the Social domain, where individuals with hypertension scored approximately 2.49 points lower than those without (*β* = −2.49, SE = 0.61, 95% CI: −3.70 to −1.29, *p* < 0.001). There were.

**FIGURE 4:**
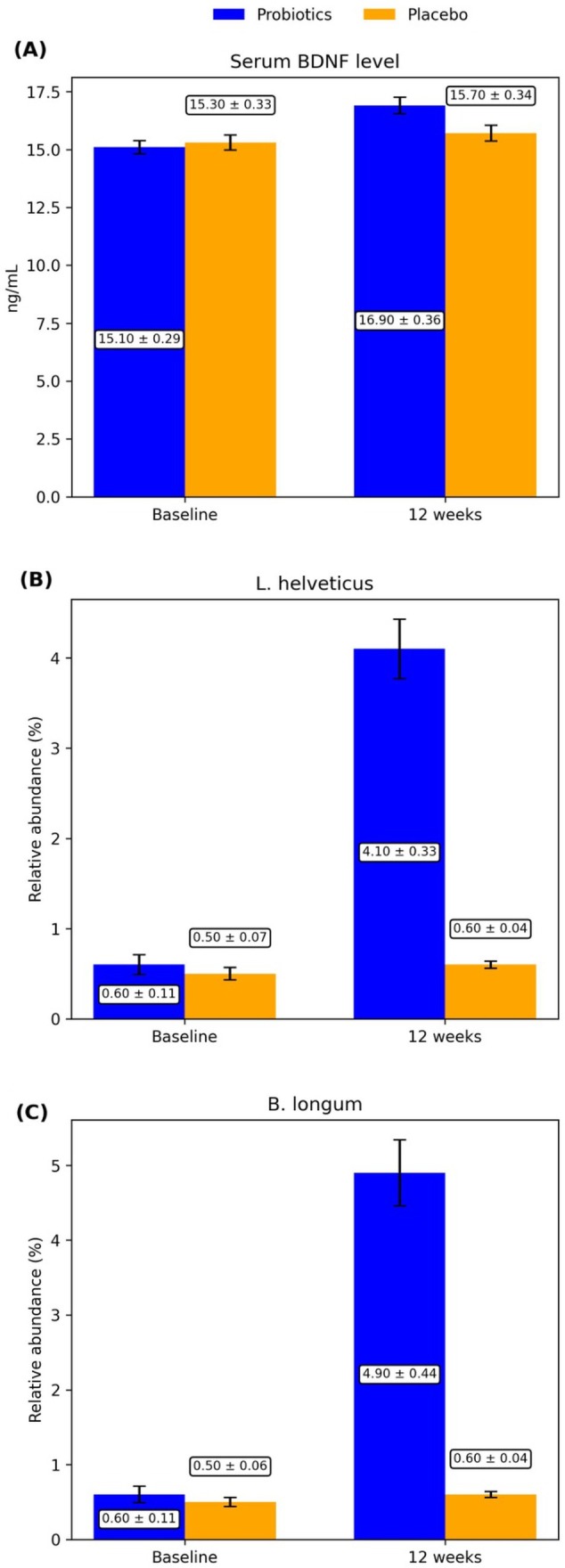
Changes in the serum brain‐derived neurotropic factor level (A) and the relative abundance of *Lactobacillus helveticus* (B) and *Bifidobacterium longum* (C) in the two groups at different timepoints (per‐protocol analysis). The results are represented by the mean ± standard error of the mean.

At baseline, serum BDNF levels were comparable between the probiotics and placebo groups. At 12 weeks, the probiotics group demonstrated a significant elevation in BDNF levels compared to the placebo group. The between‐group differences showed significantly increased serum BDNF level in the probiotics group (*p* = 0.04) at 12 weeks compared to baseline, but not in the placebo group (Figure 4A). In the probiotics‐treated patients, the fecal abundance of *L. helveticus* (Figure 4A) and *B. longum* (Figure 4B) rose significantly from baseline to 12 weeks (approximately 8–10‐fold increases, *p* < 0.001), whereas no significant change occurred in the placebo group. No serious adverse psychiatric events were reported. Mild gastrointestinal disturbances (e.g., abdominal fullness, bloating, and constipation) were noted during the first 6 weeks among seven patients, exclusively in the probiotics group.

## 4 Discussion

In this pilot PRODG trial, adjunctive probiotic therapy produced modest but meaningful reductions in depressive and anxiety symptoms compared with placebo, while both groups demonstrated substantial overall improvement across 24 weeks. Although mood symptoms improved, probiotics did not confer clear additional gains in QoL, suggesting that symptomatic change may not immediately translate into functional recovery, or that longer follow‐up may be required. Importantly, the probiotic group exhibited significantly increased serum BDNF levels at 12 weeks and striking rises in fecal *L. helveticus* and *B. longum* abundance, consistent with a potential gut‐brain pathway involving neurotrophic signaling. These biological changes parallel emerging evidence that microbiota modulation may influence neural plasticity and stress responses. The intervention was generally well tolerated, with only mild and transient gastrointestinal effects reported, and no serious psychiatric adverse events. Taken together, our findings support probiotics as a promising adjunct rather than a replacement for standard treatment, with benefits that appear modest, biologically plausible, and clinically safe.

Apart from these primary intervention effects, some clinical covariates were identified as having a significant correlation with outcome measures, adding complexity to the observation of treatment response among this group of patients. A positive correlation was found between escitalopram equivalent antidepressant dose and levels of depressive symptomatology, suggesting that underlying confounding by indication may have contributed towards the perceived improvement in MADRS and GAD‐7 scores in an escitalopram equivalent antidepressant dose‐related manner—that is, patients estimated to have more severe depression were prescribed more antidepressant medication. Moreover, benzodiazepine use correlated strongly with high levels of anxiety symptoms and impairment in social and physical quality‐of‐life measures, suggesting that confounding by sedative treatment may have contributed towards perceived treatment benefits in GAD‐7 outcomes in unadjusted analyses. In further elucidation of predominant underlying medical illnesses among this clinical population, hypertension independently correlated with impaired social quality‐of‐life measures, suggesting that significant impact from underlying cardiometabolic comorbidity contributes towards the affective burden experienced by these patients with coexisting psychiatric illness.

Our findings align with the literature. The modest improvement observed in the placebo group aligns with the trajectories described in previous studies, where 44%–46% adults with depression and anxiety disorders treated with selective serotonin reuptake inhibitors and serotonin and norepinephrine reuptake inhibitors exhibited clear symptom improvement (MADRS and GAD‐7 scores) over 12 weeks [35, 36]. Our findings in the intervention group align with growing evidence supporting probiotics in depression treatment. A 2023 meta‐analysis of 13 randomized controlled trials (*n* = 786) confirmed that probiotics significantly improve depressive symptoms, especially in mild‐to‐moderate cases [37]. Another recent review concluded that most trials published between 2014 and 2023 show a positive effect of probiotics on depression and anxiety [38]. Notably, the *L. helveticus* and *B. longum* strains used in our study were tested by Heidarzadeh‐Rad et al. [39], who observed improvements in depressive symptoms and serum BDNF levels. Our results reflect similar patterns, suggesting these strains exert antidepressant effects, possibly via neurotrophic mechanisms. Another pilot randomized controlled trial also demonstrated that probiotics, as adjuncts to antidepressants, improved depression scores more than a placebo [40]. Similarly, Schaub et al. showed clinical and microbiome improvements in depressed patients taking high‐dose probiotics [41]. Increased abundance of *Lactobacillus* correlated with symptom relief, mirroring our findings. However, some trials have reported negative results. Romijn et al. found no benefit in subclinical populations [42], highlighting how baseline symptom severity and host microbiota may influence results. In the geriatric cohort, the effect was more prominent, consistent with studies targeting clinical depression [43, 44].

Regarding cognitive functions, consistent with the literature, our findings suggest that cognitive functioning remains largely unaltered despite clinical gains in depressive and anxiety symptoms with standard antidepressant treatment [45]. The elevation in serum BDNF observed in both groups resonates with earlier findings where conventional antidepressant treatments were shown to upregulate BDNF levels, thereby reinforcing the plausibility of a shared neurobiological pathway underpinning mood improvement [46, 47]. Finally, the improvements in physical and psychological domains of WHOQOL‐BREF scores in our study are consistent with the literature, which demonstrated that patients with moderate depression could experience significant increases in the QoL within 8 weeks of initiating antidepressant therapy [48]. While many probiotic trials focus on mood scores, some, like Dehghani et al., report improvements in well‐being and functioning [45]. We echo those findings. In contrast, we did not find significant changes in cognition (MoCA). Cognitive changes also likely demand extended follow‐up or multi‐domain interventions. Romijn et al. also found no cognitive benefit in their study [42]. Dehghani et al. suggest that cognitive reactivity or memory domains may show change with longer durations [45].

Our study reinforces the gut‐brain axis hypothesis. Probiotics may act via microbial metabolite production, neuroinflammatory modulation, and neurotransmitter regulation [6, 7, 49]. Successful colonization of *L. helveticus* and *B. longum* in our study supports their biological role. These strains have been shown to reduce cortisol and cytokines in animal and human studies [50]. We also observed a significant increase in BDNF, a neurotrophin known to be reduced in depression and elevated with successful therapy. This supports findings by Heidarzadeh‐Rad et al. [39] and others [45], where probiotics increased BDNF and correlated with mood improvement. Probiotics might suppress pro‐inflammatory cytokines, indirectly enhancing BDNF expression. Metabolites like butyrate also stimulate BDNF via histone modification. Moreover, tryptophan metabolism shifts (e.g., reduced kynurenine) could play a role, as seen in studies by Rudzki et al. [51] Thus, our convergence of improved symptoms and elevated BDNF supports the hypothesis of gut‐mediated neuroregulation.

This study has high relevance for geriatric mental health in India, where depression in older adults is under‐recognized and undertreated [52, 53]. Barriers like stigma and medication side effects discourage formal psychiatric care [53]. The improvements in depressive and anxiety symptoms, along with psychological quality‐of‐life gains observed in our trial, are particularly important in the Indian geriatric context, where it was reported that social and economic disadvantages substantially impair QoL in older adults—factors that often exacerbate depression and anxiety symptoms in this vulnerable population [54]. A safe, culturally acceptable intervention such as probiotics could serve as a useful adjunct or, eventually, a first‐line approach in mild‐to‐moderate cases. Curd and other fermented foods already form part of traditional diets, offering an opportunity for community‐level implementation. In line with this, an Indian study showed that probiotic curd or capsules improved QoL in depressed individuals within 4 weeks [55]. Our study adds randomized trial evidence to this domain. Given India's rapidly aging population, probiotics may offer a scalable, low‐risk tool to support mental health in older adults, especially where access to psychiatric care is limited [54, 56].

The strength and novelty of our study are the inclusion of a homogenous cohort of older patients with moderate depression and the use of a robust study design with objective assessments (serum BDNF level and fecal microbiota profiling). The sustenance phenomenon of antidepressant and anxiolytic effects in our geriatric patients, even 3 months after withdrawal of probiotics, is also a novel finding with major potential to open possibilities of new therapeutic guidelines. The stigma of mental health intervention and medication adherence is a real‐world problem. Probiotic co‐therapy at the beginning of SOC may significantly improve outcomes even in a scenario of poor drug adherence.

There were several limitations in our study. First, a number of placebo group participants demonstrated worsening of depressive symptoms despite continuing to receive standard antidepressant therapy. This implies either that SOC was not adequately optimized in this trial context or that some participants were inherently non‐responsive. This pattern weakens the comparability of the two arms and reduces confidence in the magnitude of the observed probiotic‐placebo differences. Second, there was a large heterogeneity in the prescription of antidepressants among participants. Both the choice of agent and the titrated doses varied according to individual clinician judgment. Such variability mirrors real‐world practice but inevitably introduces uncontrolled confounding. Third, the sample size was small, and the study was not powered. The planned sample size of 80 could not be attained within the stipulated timeframe. There was a high rate of attrition (> 50% by 21 weeks). This could be because of the difficulty with adherence for cognitive, physical, or social reasons. However, there were no significant differences in the baseline MADRS score and the duration of depression between participants who continued in the trial and who were lost to follow‐up at 12 weeks (primary outcome). Such studies in a community set‐up and enforcing a stricter follow‐up would yield more robust results. Fourth, microbiota analysis was done only in a subset. Inflammatory markers or neurotransmitters were not assessed, which could have strengthened mechanistic conclusions. Fifth, dietary intake of curd or yoghurt, which was not quantified or monitored, could have influenced the outcome across both groups. Finally, serum BDNF level estimation or fecal microbiota profiling was not performed at 24 weeks, which could have given more mechanistic insights.

Collectively, these methodological limitations—the unexpected deterioration in the placebo arm despite antidepressant use, the nonuniform antidepressant regimens, and the exceptionally high drop‐out rate—suggest that credibility and stability for the results are lower than the initial analysis might suggest. The results should therefore be interpreted cautiously, and replication in larger, better‐controlled studies is necessary. Future studies should include patients with common geriatric comorbidities. A larger trial with longer follow‐up is needed to confirm efficacy, explore durability, and refine the therapeutic model. The optimum combination of strains of probiotics and their strength and dose also needs to be explored.

## 5 Conclusion

In this pilot PRODG trial, adjunctive probiotics produced modest symptom benefits over placebo and increased BDNF and beneficial gut species but did not improve QoL beyond usual care. Overall, probiotics appear safe, biologically plausible, and promising as supportive therapy for patients with depression. These findings support a growing body of literature on probiotics and hold special promise for low‐resource settings like India. However, the interpretation of these findings is constrained by substantial dropout and marked heterogeneity in antidepressant regimens across participants, which reduces internal validity. Future large‐scale trials with standardized treatment protocols and improved retention strategies are essential to confirm the reproducibility and reliability of these preliminary results.

## Author Contributions

A.G. and S.D. conceived the original idea. Further development of the study plan: P.S., P.C., A.M., G.K., J.J.C., and A.V.; patient recruitment, treatment, and follow‐up: P.S., P.C., P.K., and A.C.; patient assessment and data collection: K.S.R., R.P., and S.K.; study co‐ordination: S.M., S.S., M.B., M.M., K.B., and S.M.; statistical analysis: P.S., M.K., and S.D.; result interpretation: P.S., P.C., I.S., A.G., and S.D.; manuscript writing: A.G. and S.D.; overall supervision and guidance: I.S., A.K.D., A.S., and A.C. Funding acquisition as Principal Investigator: S.D.

## Funding

This study was funded by the Department of Health Research, Ministry of Health and Family Welfare, Government of India, through the Grant‐in‐Aid program for inter‐sectoral convergence and coordination to support health research (file no. R.11014/06/2023‐GIA/HR). The open access funding was provided by Karolinska Institutet, Stockholm, Sweden.

## Ethics Statement

The PRODG trial was approved by the ethics committees of the two study sites. The trial was registered with the Clinical Trials Registry of India (CTRI/2024/01/061173). Written informed consent was obtained. The study was performed following the Indian Council of Medical Research National Ethical Guidelines for Biomedical and Health Research Involving Human Participants, 2017. All patients provided written informed consent before participating in the study.

## Conflicts of Interest

The authors declare no conflicts of interest.
